# Topics of the Month

**Published:** 1892-12

**Authors:** 


### TOPICS OF THE MONTH.

THE Southern Surgical and Gynecological Association held its fifth annual meeting in Louisville, Ky., November 15, 16, and 17, 1892, and it may be stated that in all respects it was a creditable gathering of prominent medical men, principally from the South, who, during the sessions, contributed to make a volume that will be a valuable addition to the medical literature of the year.

It is not necessary at this time to speak in detail of this meeting, an account of which will go to the medical journals and also be published in the Transactions of the Association ; but it is pertinent to remark that for genuine hard work for three days in succession, this meeting deserves to rank among the busiest and best of all the medical gatherings in the United States held during the year 1892.

The Chairman of Committee of Arrangements, Dr. L. S. McMurtry, planned and executed a complete system of caring for the visitors, both in a scientific and social way, so that while the meeting went on with regularity and precision, there were yet sufficient social features to afford mental relief, though these did not interfere at all with the work of the Association.

Dr. W. E. B. Davis, the Secretary, exhibited great skill and discretion in preparing a valuable programme and in handling it well on the floor, so that no time was sacrificed and no member disturbed by being overlooked or pushed aside.

The election of Dr. Bedford Brown, of Alexandria, Va., to the presidency for the ensuing year was a fitting tribute to a faithful and industrious member, who has contributed to the scientific interest of every meeting, and who has attended, in person, each and all of them. The Association honored itself in choosing Dr. Brown as its presiding officer for the coming year.

The next meeting will be held in New Orleans, beginning on the second Tuesday of November, 1893, and it is expected that many members will be interested in visiting the Crescent City with their wives and families ; hence, the attendance will be large. When the volume of Transactions for 1892 is issued, it will furnish interesting reading that will be sought eagerly by surgeons and gynecologists as well as general practitioners all over the land.

---

A Section of Railway Surgery of the Pan-American Medical Congress has been organized, with Dr. C. W. P. Brock, of Richmond, Va., as Executive President. A full list of officers has been provided for each of the constituent countries. At the eleventh annual meeting of the Wabash Railway Surgical Association—the first organization of the kind—Dr. C. B. Stemen, of Fort Wayne, was, by unanimous resolution, requested to prepare a paper on Organized Railway Surgery, and read the same before the Section on Railway Surgery of the Pan-American Medical Congress. At the same meeting, Dr. Hal C. Wyman, of Detroit, offered the following, which was unanimously adopted :

*Resolved,* That each member of this Association solicit his Congressman to interest himself in legislation in favor of the Pan-American Medical Congress.

---

A Bureau of Hygiene and Sanitation has been organized, to prepare a collective exhibit illustrative of the present condition of sanitary science, under the auspices of the Department of Liberal Arts of the World's Columbian Exposition. The aim of this bureau will be to show, as nearly as possible, the position in which the theory and practice of hygiene stands at the present day, and the superintendent, Dr. F. W. Brewer, has issued a circular of fourteen octavo pages relating to its functions and the executive details thereof. In this circular Dr. Brewer says :

It is hoped that the universities and colleges, the boards of health, State and municipal, the societies having hygiene and sanitation as their key-notes, the scientists, the physicians, the manufacturers, and the public generally will cordially coöperate in the endeavor to make the exhibition worthy of the science and of our country.

There is ample opportunity here for sanitarians, public health officers, boards of health, and all men interested in the prevention of disease to unite in contributing to the success of what ought to be one of the most useful departments of the Columbian Exposition to be held in Chicago next year. We hope that some of our local sanitarians will interest themselves in this bureau and will contribute something that will indicate that Buffalo is well to the fore in all questions relating to hygiene, sanitation, and public health.

---

One Way to Advertise.—A sample copy of a religious paper was received, presumably by a number of Buffalo physicians. Beneath the address label was stamped in large letters: "Sample Copy for Himself and His Most Devout Convalescing Patient."

---

In Dr. Landon Carter Gray's forthcoming Text-Book of Nervous and Mental Diseases, American physicians are promised a clear statement of a department of medicine of peculiar importance to them. The task assumed by the author is one of exceptional difficulty. It would have been comparatively easy to gather abundant literature upon a subject of such enormous growth, but to weigh, select, and condense requires exceptional qualities of mind. In a word, Dr. Gray has undertaken to provide students and practitioners with a compact volume containing a working knowledge of neurology, space being principally devoted to the diagnosis and treatment of all diseases belonging to this department. The series of illustrations will be largely original and singularly rich. The work is assured of the highest position as an authority for the physician and as a text-book for the student.

---

The U. S. Pharmacopeia, 1890, which will be published during 1893, adopts in great measure the metric system of weights and measures; this will doubtless create much confusion in the minds of physicians and druggists, and lead to many misunderstandings and errors. In order to provide a guide to the proper dosage, etc., Dr. George M. Gould, author of the New Medical Dictionary, has prepared a very complete table of the official and unofficial drugs, with doses in both metric and English systems; this table is to be published in P. Blakiston, Son & Co.'s Physicians' Visiting List for 1893, together with a short description of the metric system.

